# Macular dystrophy associated with the mitochondrial DNA A3243G mutation: pericentral pigment deposits or atrophy? Report of two cases and review of the literature

**DOI:** 10.1186/1471-2415-14-77

**Published:** 2014-06-06

**Authors:** Alejandra Daruich, Alexandre Matet, François-Xavier Borruat

**Affiliations:** 1 Department of ophthalmology, Jules Gonin Eye Hospital, Fondation Asile des aveugles, University of Lausanne, Avenue de France 15, Case postale 133, CH-1000 Lausane7, Switzerland

**Keywords:** MELAS, MIDD, Macular dystrophy, A3243G

## Abstract

**Background:**

The A3243G point mutation in mitochondrial DNA (mtDNA) is associated with MELAS (mitochondrial encephalomyopathy with lactic acidosis and stroke-like episodes) and MIDD syndromes (maternally inherited diabetes and deafness). Both MELAS and MIDD patients can present with visual symptoms due to a retinopathy, sometimes before the genetic diagnosis is made.

**Case presentation:**

Patient 1: 46 year-old woman with diabetes mellitus and hearing loss was referred for an unspecified maculopathy detected during screening evaluation for diabetic retinopathy. Visual acuity was 20/20 in both eyes. Fundus examination showed bilateral macular and peripapillary hyperpigmented/depigmented areas.

Patient 2: 45 year-old woman was referred for recent vision loss in her left eye. History was remarkable for chronic fatigue, migraine and diffuse muscular pain. Visual acuity was 20/20 in her right eye and 20/30 in her left eye. Fundus exhibited several nummular perifoveal islands of retinal pigment epithelium atrophy and adjacent pale deposits in both eyes.

Retinal anatomy was investigated with autofluorescence, retinal angiography and optical coherence tomography. Retinal function was assessed with automated static perimetry, full-field and multifocal electroretinography and electro-oculography. Genetic testing of mtDNA identified a point mutation at the locus 3243.

**Conclusion:**

Observation of RPE abnormalities in the context of suggestive systemic findings should prompt mtDNA testing.

## Background

The A3243G mitochondrial DNA (mtDNA) point mutation has been associated with a wide range of clinical manifestations, including MELAS syndrome (mitochondrial encephalomyopathy with lactic acidosis and stroke-like episodes) and MIDD syndrome (maternally inherited diabetes and deafness) [1,2].

The ocular findings of MELAS include pigmentary retinopathy, external ophthalmoplegia, ptosis and posterior subcapsular cataract [3,4]. The main ocular feature in MIDD syndrome is a pigmentary retinal dystrophy also described as a macular pattern dystrophy [5,6]. The retinopathies associated with both syndromes have heterogeneous presentations with variable degrees of retinal pigment epithelium atrophy and hyperpigmentation at the posterior pole [3,5-7].

De Laat et al. proposed a classification system based on 4 grades: fine pigment abnormalities (grade 1), yellowish or mildly pigmented deposits (grade 2), chorioretinal atrophy outside the fovea (grade 3) and atrophy affecting the fovea (grade 4) [8].

Rath et al. described two distinctive phenotypes of retinopathy [9]. The first phenotype is characterized by a discontinuous perifoveal atrophy that assumes a circumferential distribution. With time, the areas of atrophy will coalesce into a ring of pericentral atrophy. Occasionally, pale deposits can be present at the level of the retinal pigment epithelium (RPE), the RPE can exhibit a granular appearance, and subretinal pigment clumping can be found adjacent to the areas of atrophy.

The second phenotype is consistent with a pattern dystrophy. It includes granularity of the RPE, pale deposits and pigment clumping at the level of the RPE, without any significant atrophy.

We report two patients with the mtDNA A3243G point mutation who each presents one of the previously described retinal phenotypes.

## Case presentation

### Patient 1

**Figure 1:**
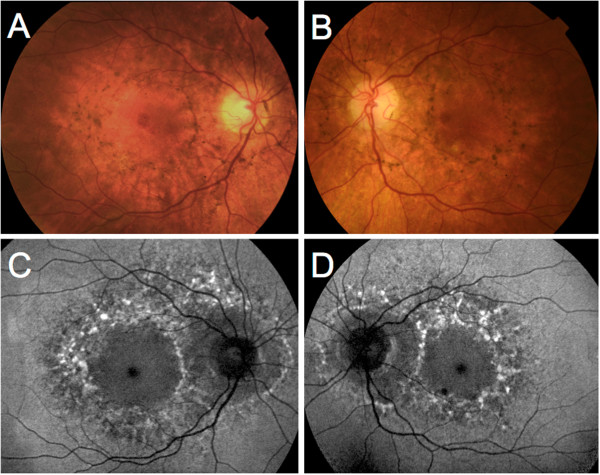
**Color fundus photograph and fundus autofluorescence of Patient 1. A**, **B**, Color fundus photograph of patient 1. Note the hyperpigmented lesions surrounding the macula and the optic disc, associated with depigmentation areas of the retinal pigment epithelium. **C**, **D**, Fundus autofluorescence of the same patient. The hyperpigmented spots correspond to mainly increased autofluorescence, whereas the hypopigmented spots correspond a decreased granular autofluorescence signal.

**Figure 2:**
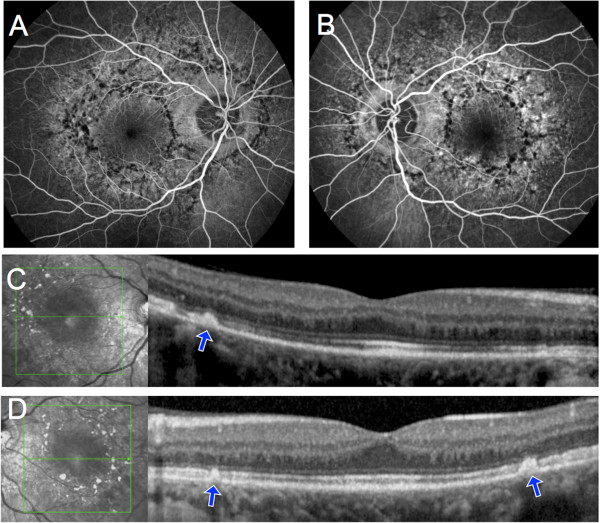
**Fluorescein angiography and SD-OCT of Patient 1. A**, **B**, Fluorescein angiography showed blockage of background fluorescence in the areas of hyperpigmentation and hyperfluorescence due to retinal pigment epithelium window defects in the depigmented areas. **C**, **D**, On SD-OCT scans taken through the planes indicated by the green arrows, hyperpigmented areas corresponded to a hyperreflective dome-shaped lesion that seemed to originate from the retinal pigment epithelium (blue arrows).

**Figure 3:**
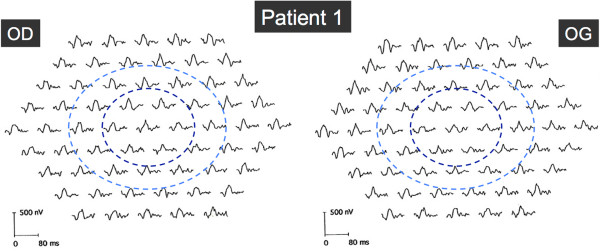
**Multifocal ERG in Patient 1.** Multifocal ERG showed a moderate and diffuse depression of amplitudes in both eyes. Dark and light blue rings indicate the 5° and 10° circles centered by the fovea, respectively.

A 46 year-old woman was referred for an unspecified maculopathy detected during screening evaluation for diabetic retinopathy. Insulin-dependent diabetes mellitus was diagnosed at age 20. The patient had bilateral sensorineural hearing loss since age 41 and was wearing a hearing aid. Migraine was present since age 26. Family history revealed that her mother died from stroke at age 43. Five out of six maternal aunts and uncles were affected by diabetes mellitus and/or hearing loss.Best-corrected visual acuity was 20/20 in both eyes. Fundus examination revealed bilateral hyperpigmented lesions surrounding the macula and the optic disc, associated with depigmentation areas of the RPE. Fundus autofluorescence (Heidelberg Retina Angiograph; Heidelberg Engineering, Heidelberg, Germany) was characterized by a diffuse speckled appearance of the macula (Figure 1). Fluorescein angiography displayed a mottled appearance of the posterior pole with adjacent areas of hyper- and hypofluorescence, indicating defects of the RPE and pigment deposits, respectively. Spectral-Domain Optical Coherence Tomography (SD-OCT, Spectralis, Heidelberg Engineering, Heidelberg, Germany) scans taken through the hyperpigmented lesions showed a hyperreflective dome-shaped change that seemed to originate from the RPE (Figure 2).Results from automated static perimetry (Octopus 300, program G1, Haag-Streit, USA), scotopic and photopic full-field electroretinography (ERG) were normal in both eyes. Multifocal ERG (mfERG) showed a moderate and diffuse depression of potentials in both eyes (Figure 3). Electro-oculogram (EOG) was normal with an Arden ratio of 2.2. Magnetic resonance imaging (MRI) of the brain was unremarkable. Genetic testing of mtDNA identified a point mutation at the locus 3243 with 35% heteroplasmy in leucocytes.

### Patient 2

A 45 year-old woman was referred for recent vision loss in her left eye in the context of unusual retinal findings. She had short stature and hearing difficulties were noticed since age 28. There was no history of diabetes mellitus. Personal history revealed chronic fatigue, migraine, diffuse muscular pain and episodes of vomiting for the past 3 years. She was also treated for a depressive state.

**Figure 4:**
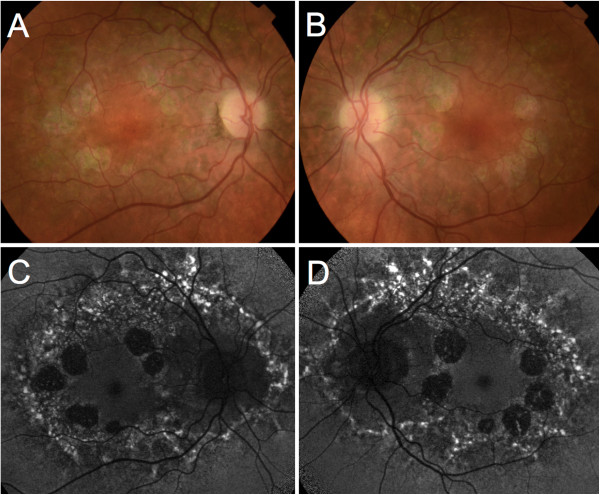
**Color fundus photograph and fundus autofluorescence of Patient 2. A**, **B**, Color fundus photograph of patient 2. Note the circumferential perifoveal patches of atrophy and the adjacent pale deposits. **C**, **D**, Fundus autofluorescence of the same patient showed decreased autofluorescence signal inside the atrophic areas, and increased signal in the areas corresponding to the deposits, which surrounded both the macula and optic disc.

**Figure 5:**
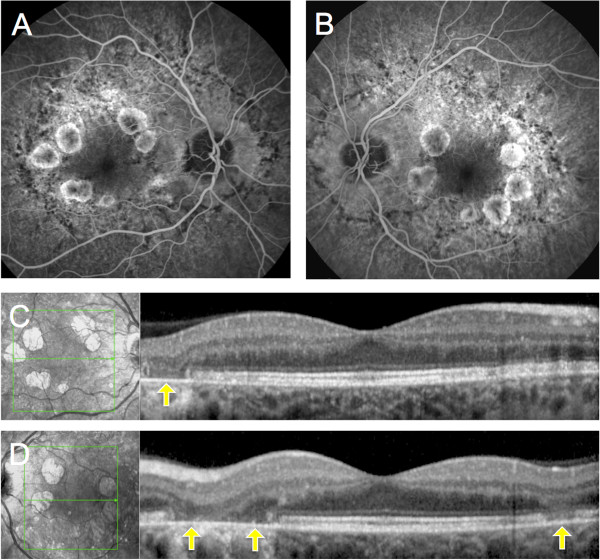
**Fluorescein angiography and SD-OCT of Patient 2. A**, **B**, Fluorescein angiography of the patient 2, showed window defects in the area of the atrophy and a blocking effect by the pale deposits. **C**, **D**, On horizontal OCT scans taken through the planes indicated by the green arrows, the outer retina was markedly attenuated in the zones of chorioretinal atrophy (yellow arrows).

**Figure 6:**
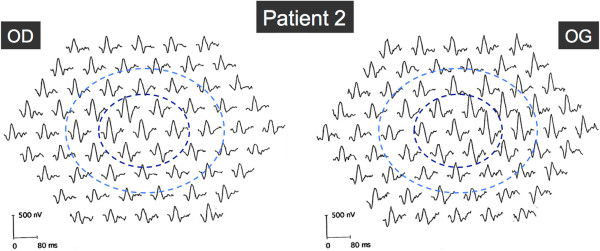
**Multifocal ERG in Patient 2.** Multifocal ERG showed slightly decreased amplitudes in a pericentral zone outside the central 5°, in both eyes. Dark and light blue rings indicate the 5° and 10° circles centered by the fovea, respectively.

Family history revealed that her mother suffered from both diabetes mellitus and hearing loss. Her sister was diagnosed with a psychiatric disorder and suffered from recurring seizures.Best-corrected visual acuity was 20/20 in her right eye and 20/30 in her left eye. Fundus examination revealed several circumferential perifoveal islands of atrophy and adjacent pale deposits in both eyes. Autofluorescence was decreased inside the atrophic areas, and was increased in the areas corresponding to the deposits. The retina surrounding the atrophic patches demonstrated a speckled autofluorescence (Figure 4). Fluorescein angiography showed window defects in the area of the atrophy and a blocking effect by the pale deposits. On SD-OCT the central foveal anatomy was relatively preserved, with parafoveal zones of attenuation of the outer layers corresponding with chorioretinal atrophy zones (Figure 5).Results from automated static perimetry (Octopus 300, program G1, Haag-Streit, USA) and scotopic and photopic full-field ERG were within normal limits in both eyes. Multifocal ERG showed slightly decreased potentials in a pericentral zone in both eyes (Figure 6). Electro-oculogram (EOG) was normal with an Arden ratio of 2.7. Brain MRI was normal. Genetic testing of mtDNA extracted from a muscular biopsy identified a point mutation at the locus 3243, and demonstrated 71% of heteroplasmy. A diagnosis of MELAS was given.

## Discussion

We report two patients harboring the mtDNA A3243G point mutation who exhibited two different retinal phenotypes. Namely, the degree of RPE changes was significantly different between our two patients.

According to existing studies, whether the various retinal findings associated with this mtDNA point mutation are different evolutive stages or correspond to distinct phenotypes is not fully understood.

Diagnosis suspicion of MIDD or MELAS relies on the association of characteristic retinal and systemic abnormalities, in a context of maternal inheritance. Yet, mutation carriers can present a wide range of retinal phenotypes, and sometimes minimal or absent systemic findings. As a consequence, characterization of retinal features is crucial for optimal diagnosis and management. Main differential diagnoses include: late-onset Stargardt disease, multifocal pattern dystrophy, central areolar choroidal dystrophy and age-related macular degeneration.

Late-onset Stargardt disease presents in patients over 50 years of age with hyperautofluorescent flavimaculatus flecks and relative foveal and peripapillar sparing [10]. Flecks appear on SD-OCT as hyperreflective irregular thickening of the RPE similar to the deposits seen in mtDNA A3243G retinopathy, as in Patient 2. However, these patients exhibit a characteristic ‘dark choroid’ on fluorescein angiography and have recessive autosomal inheritance of ABCA4 mutations.

In multifocal pattern dystrophy simulating Stargardt disease, size and distribution of flecks are similar to Stargardt disease; yet these patients always display foveal involvement ranging from yellow-grayish deposits to atrophic lesions, and do not have a ‘dark choroid’ on fluorescein angiography [11]. Central areolar choroidal dystrophy should be considered because of the wide and well-circumscribed central patch of atrophy, that usually starts outside the fovea inside a hypopigmented area, and widens until it involves the whole macular area. Flecks are absent, but drusen-like lesions may be observed on fundus examination [12]. Both multifocal pattern dystrophy and central areolar choroidal dystrophy are associated with mutations of the PRPH2 gene with autosomal dominant inheritance.

Finally, age-related macular degeneration (AMD) is the most frequent cause of atrophic maculopahy in older age and presents drusen that can resemble deposits seen in patients with mtDNA A3243G mutations. In addition, earing difficulties and diabetes are also more frequent among older patients. However, in AMD drusen are located beneath the RPE on SD-OCT, are less hyperautofluorescent, more coalescent and located more centrally than in mtDNA A3243G retinopathy. Moreover, in AMD atrophy emanates either from these drusen or from an RPE detachment or tear.

Mitochondrial disorders lead to a decreased production of ATP. The failure to meet the cellular energetic needs will result in a clinical specific phenotype depending on the affected cells. The clinical spectrum of mitochondrial disorders is broad. However, tissues with a high metabolic demand are typically affected [13]. An outer retinopathy could result from a primary dysfunction of the photoreceptors, a primary dysfunction of the RPE, or a dysfunction of both retinal layers. The RPE is responsible for maintenance of photoreceptor function through continuous phagocytosis, recycling, and nourishment of outer segments. The photoreceptors are constantly degraded and resynthesized, and contain clusters of mitochondria within their inner segments [14]. Whether these two cell layers are equally susceptible to the effects of the A3243G mtDNA mutation, or whether the photoreceptor damage is a consequence of the RPE disease, is not known. In Patient 1, results of mfERG showed a diffuse but moderate decrease in amplitudes, whereas Patient 2 exhibited an annular slight decrease in amplitudes, outside the central 5° in which amplitudes were normal. As mfERG represents mostly a photopic response, we can presume that Patient 1 suffered from a moderate but diffuse central cone dysfunction, roughly corresponding to the macular lesion visible on autofluorescence, whereas the annular decrease of mfERG in Patient 2 resulted from the pericentral zones of atrophy. Abnormalities in mfERG suggesting a damage in the cone photoreceptor outer segments have already been described in MIDD patients [15].

RPE function can be assessed by EOG. The two patients described herein have normal EOG. Abnormal EOGs have been reported in approximately a third of patients, and seem more frequent in patients with advanced disease. Remarkably, reported Arden ratios are only slightly diminished [5,8]. These contradictory findings do not provide further insight into the possible etiology of the disease but indicate that the function of the RPE is either normal or slightly diminished in patients with mtDNA A3243G mutations.

Rummelt et al. described retinal histopathologic findings in a patient with MELAS and severe retinal dystrophy [4]. At the posterior pole, RPE cells were depleted of apical microvilli, melanosomes and phagosomes, and contained abnormal mitochondria. Mitochondria in the inner photoreceptor segments were also abnormal and the outer segments were atrophic. Retinal pigment epithelium and photoreceptor cells in the retinal periphery were unaffected.

The two cases presented here illustrate that the severity of systemic and ocular phenotypes could be related. Indeed, both retinal and systemic manifestations were milder in Patient 1 than in Patient 2. Patient 1 might then represent a milder form of A3243G-associated disease.

Variability of phenotypes associated with mtDNA mutations is well recognized [13]. Such a variation in the phenotypic appearance is presumed to be a consequence of the variable proportions of mutated and normal mtDNA between patients, but also between different tissues within a single individual. This is a characteristic feature of mitochondrial disease known as heteroplasmy. In this study, heteroplasmy was higher in Patient 2 than in Patient 1. However, the direct comparison of these percentages is not appropriate since they originate from muscle and leucocytes, respectively.

Latvala et al. evaluated 26 patients with A3443G mtDNA mutation and correlated the presence of RPE abnormalities to the severity of the systemic phenotypes [16]. They showed that both the severity of the systemic disease and the degree of mutant heteroplasmy in muscle correlate positively with RPE abnormalities. The risk of RPE abnormalities was almost 3-fold higher in patients who harbored >68% mutant heteroplasmy in muscle compared to those below these limits. Hence, RPE abnormalities appear to be a retinal manifestation associated with more severe disease phenotypes.

De Laat et al. analyzed 25 patients with the A3243G mutation and retinal abnormalities [8]. The retinal abnormalities were classified into four grades of severity, from fine pigment abnormalities to profound chorioretinal atrophy. The severity of retinal dystrophy was not correlated with heteroplasmy levels measured in the urinary epithelial cells, leukocytes and buccal saliva. There were no correlation between the severity of the retinal dystrophy and the overall disease involvement. These results are in contradiction with the study by Latvala et al. in which, remarkably, heteroplasmy was assessed from muscle biopsies in all patients.

Smith et al. found that among 13 subjects with MIDD, pigmentary retinal dystrophy and abnormal ERG findings were more frequent in patients over 40 years of age and in those with diabetes duration longer than 5 years [5]. In this study the presence of systemic features of A3243G disease in all patients with retinal dystrophy, and the comparatively mild phenotypes of two subjects with no retinal disease, also support the hypothesis of a correlation between systemic and ocular phenotypes as a consequence of heteroplasmy. However, age and duration of diabetes were not correlated with severity of the retinal pigment abnormalities.

Further, Massin et al. analyzed 33 patients with MIDD, and found no significant correlation between severity of macular dystrophy and patients’ age or duration of diabetes (Table 1) [6].

**Table 1:** RPE changes in patients with A3243G mtDNA point mutation and correlation with systemic disease

**Authors**	**Total number of patients**	**Systemic phenotype**	**Mean age (years)**	**Sex ratio (F/M)**	**Number of patients with RPE changes (%)**	**Correlation of RPE changes with:**		
						**Systemic disease**	**Age**	**Hetero-plasmy**
Laat et al. [8]	29	24: MIDD	46	20/9	25 (86%)	No	Yes	No
		4: MELAS						
		1: Cardiopathy						
Latvala et al. [16]	26	7: MIDD	44	21/5	10 (38%)	Yes	-	Yes
		10: DM						
		12: Hearing loss						
Massin et al. [6]	38	35: MIDD (33: DM)	49	22/13	31 (81.6%)	No	No	-
		3: MELAS	46	0/3				
Smith et al. [5]	13	MIDD	49	9/4	10 (77%)	No	No	-
Sue et al. [3]	14	MELAS	42	8/6	8 (57%)	-	-	-

MIDD = maternally inherited diabetes and deafness; MELAS = mitochondrial encephalomyopathy with lactic acidosis and stroke-like episodes; DM = diabetes mellitus.

Finally, characteristics of the mtDNA-A3243G-associated retinopathy are variable and must be distinguished from related findings exhibited by other retinal conditions as retinal dystrophies and AMD. Association of systemic features and maternal inheritance should contribute to sharpen the diagnosis. Yet, the correlation between retinal and systemic phenotypes is not clear. Identification of the mtDNA mutation confirms the diagnosis. However, up to 15% of false negative blood tests have been reported, which is explained by the heteroplasmy affecting mtDNA [17]. As a consequence, genetic testing should be repeated on other tissues (urinary epithelial cells, buccal mucosa or muscle) in cases of strong clinical suspicion with previous non-contributive mutation search.

## Conclusion

Variable degrees of RPE changes are observed among patients with the A3243G mtDNA mutation. The correlation of theses findings with the systemic involvement remains unclear. Observation of RPE abnormalities in the context of suggestive systemic findings should prompt mtDNA testing.

## Consents

Written informed consent was obtained from the patients for publication of this Case report and any accompanying images. A copy of the written consent is available for review by the Editor of this journal.

## Abbreviations

ERG: Electroretinography; MELAS: Mitochondrial encephalomyopathy with lactic acidosis and stroke-like episodes; MIDD: Maternally-inherited diabetes and deafness; MRI: Magnetic resonance imaging; mfERG: Multifocal electroretinography; mtDNA: Mitochondrial DNA; RPE: Retinal pigment epithelium; SD-OCT: Spectral-domain optical coherence tomography.

## Competing interests

The authors declare that they have no competing interests.

## Authors’ contributions

AD: data collection, analysis and interpretation of data, review of the literature and manuscript draft. AM: acquisition of data and help to draft the manuscript. F-XB: acquisition of data, coordination, clinical advice and critical revisions. All authors read and approved the final manuscript.

## Pre-publication history

The pre-publication history for this paper can be accessed here:

http://www.biomedcentral.com/1471-2415/14/77/prepub
